# A clinical evaluation of gelastic and dacrystic seizures: a multicenter study

**DOI:** 10.1055/s-0042-1758755

**Published:** 2022-12-29

**Authors:** Aylin Bican Demir, Başak Yılmaz Öz, Mustafa Onur Yıldız, Bengi Gül Türk, Taner Tanrıverdi, Ahmet Bekar, Naz Yeni, İbrahim Bora

**Affiliations:** 1 Bursa Uludag University, Faculty of Medicine, Epilepsy Center, Department of Neurology, Bursa, Turkey.; 2 Istanbul University-Cerrahpasa, Cerrahpasa Faculty of Medicine, Epilepsy Center, Department of Neurology, Istanbul, Turkey.; 3 Dr Selahattin Cizrelioglu Cizre State Hospital, Department of Neurology, Hakkari, Turkey.; 4 Istanbul University-Cerrahpasa, Cerrahpasa Faculty of Medicine, Epilepsy Center, Department of Neurosurgery, Istanbul, Turkey.; 5 Bursa Uludag University, Faculty of Medicine, Epilepsy Center, Department of Neurosurgery, Bursa, Turkey.

**Keywords:** Epilepsies, Partial, Seizures, Crying, Epilepsias Parciais, Convulsões, Choro

## Abstract

**Background** Gelastic seizures are extremely rare, short-lasting, unprovoked, and uncontrollable laughing attacks. We conducted this retrospective evaluation to determine whether these symptoms, manifesting in different forms, such as cheerful laughter, laughing, smiling, and sobbing had any value in terms of etiology or localization.

**Methods** A total of 31 patients who exhibited bouts of laughing or crying and who were under follow-up between 2000 and 2019 at tertiary epilepsy centers were included in the study. Laughing seizures were divided into three groups in terms of semiology (i.e., laughter with mirth, laughter without mirth, and smile). Dacrystic seizures were accompanied by some gelastic seizures and were divided into two groups in terms of semiology (i.e., weeping loudly [motor and voice-sobbing] and crying).

**Results** Of the 27 patients with laughing seizures, 12 had seizures that manifested with smiling, 7 had seizures that manifested with laughing and mirth, and 8 had seizures that manifested with laughter without mirth. Dacrystic-gelastic seizures were observed in four patients, among whom 2 patients had crying and laughter without mirth and 2 patients had weeping loudly and laughter without mirth episodes.

**Conclusion** Gelastic and dacrystic seizures often suggest hypothalamic hamartomas, in the literature. This rare ictal behavior can originate from different cortical locations and lesions of a different nature. However, we found that gelastic seizures with smiling were a more homogenous group with regard to location in the temporal lobe, which we aimed to show by evaluating the patients included in this study.

## Resumo

**Antecedentes** Crises gelásticas são ataques de riso extremamente raros, de curta duração, não provocados e incontroláveis. Realizamos esta avaliação retrospectiva para determinar se esses sintomas, manifestando-se de diferentes formas, como riso alegre, riso, sorriso e soluço, tinham algum valor em termos de etiologia ou localização.

**Métodos** Foram incluídos no estudo 31 pacientes que apresentavam crises de riso ou choro e que estavam em acompanhamento entre 2000 e 2019 em centros terciários de epilepsia. As crises de riso foram divididas em três grupos em termos de semiologia (ou seja, riso com alegria, riso sem alegria e sorriso). As crises dacrísticas foram acompanhadas por algumas crises gelásticas e foram divididas em dois grupos em termos de semiologia (ou seja, choro alto [motor e soluçar a voz] e choro).

**Resultados** Dos 27 pacientes com crises de riso, 12 tiveram crises que se manifestaram com sorriso, 7 tiveram crises que se manifestaram com riso e alegria e 8 tiveram crises que se manifestaram com riso sem alegria. Crises dácristico-gelásticas foram observadas em quatro pacientes, sendo 2 pacientes com choro e riso sem alegria e 2 pacientes com choro alto e riso sem alegria.

**Conclusão** Crises gelásticas e dacrísticas frequentemente sugerem hamartomas hipotalâmicos, na literatura. Este comportamento ictal raro pode ter origem em diferentes localizações corticais e lesões de natureza diversa. No entanto, verificamos que as crises gelásticas com sorriso foram um grupo mais homogêneo quanto à localização no lobo temporal, o que buscamos evidenciar avaliando os pacientes incluídos neste estudo.

## INTRODUCTION

Gelastic seizures are extremely rare, short-lasting, unprovoked and uncontrollable laughing attacks. 1 Gelastic and dacrystic seizures are classified as focal onset and nonmotor emotional seizures according to the 2017 ILAE classification. 2 Gelastic and dacrystic seizures may be isolated, but, frequently, they are embedded in a seizure with a series of symptomatology including automatisms. 3 Although gelastic seizures do not originate from a certain anatomical region according to recent studies, they can originate from the temporal, parietal or frontal lobe or from hypothalamic hamartomas originating primarily from the hypothalamus; non-lesional cases have also been reported. 3 4 Laughing and crying can manifest in different forms. We conducted this retrospective evaluation to determine whether the different manifestations of these symptoms, such as cheerful laughter, laughing, smiling, and sobbing, had any value in terms of etiology or location.

## METHODS

The present study included 31 patients from the Department of Neurology of the Epilepsy Centres of Bursa Uludag University Faculty of Medicine and the Department of Neurology of the Istanbul Cerrahpasa University Faculty of Medicine who exhibited bouts of laughing or crying and underwent video electroencephalography (EEG) monitoring between 2000 and 2019. Both universities are tertiary centers that employ epileptologists and are equipped with long-term video-EEG monitoring units. Ethical approval for this study was received from the Bursa Uludag University Clinical Trials Ethics Committee in accordance with the Declaration of Helsinki (2011-KAEK-26/253).

The data were collected retrospectively by reviewing video-EEG monitoring reports. Patients with any type of laughing or crying semiology alone or combined with other seizure symptoms and signs were included in the study. 2 Laughing seizures were divided into three groups in terms of semiology: laughter with mirth, laughter without mirth (motor and voice), and smile (motor). Dacrystic seizures were also divided into two groups in terms of semiology: weeping loudly (motor and voice-sobbing) and crying (motor). Additional symptoms other than gelastic or dacrystic semiology were also provided. Status of awareness was noted if known.

The recordings were made with 32-channel, long-term video-EEG devices. Electrodes were placed according to a 10-to-20 montage system using collodion. Activities in the ictal and interictal EEGs of the patients were classified anatomically. The activity in the interictal EEG was divided into two groups: epileptiform and non-epileptiform. Non-epileptiform activities were focal, lateralized or generalized slowing of the baseline activity. Interictal epileptiform activities and ictal EEG findings were divided into five groups according to the location and onset of ictal activity:

- Focal (limited to two adjacent electrodes)
- Regional (activity in more than two electrodes in one hemisphere)
- Generalized (generalized and simultaneous activities in both hemispheres, for instance, originating from frontal or temporal electrodes)
- Multifocal (independent focal/regional activities in both hemispheres)
- No ictal activity or only artefacts

Data from cranial magnetic resonance imaging (MRI) and positron emission tomography-computed tomography/magnetic resonance (PET-CT/MR) images were also included. Patients who had been seizing despite two proper drugs at the same time or consecutively were classified as resistant to medical treatment. 5 The type of surgery and the outcome for patients who underwent surgery were provided according to the Engel outcome. 6

## RESULTS

A total of 212 seizures of 31 patients were observed in the video-EEG room for 2 to 6 days, on average 4.3 days. Laughing and dacrystic seizures were detected in 128 (60%) of these 31 patients. Eighteen male (58%) and 13 female (42%) patients were included in the study. The mean age of the patients was 29 years (10–69). The right hand was dominant in 27 patients and the left in 4 patients. The mean age of seizure onset was 3 years (1 month–30 years). All patients, except for one, were resistant to treatment.

All 31 patients had episodes of laughing and 4 patients had episodes of crying as well as laughing. When evaluated with video-EEG monitoring, the mean duration of the seizures in 27 laughing patients was 103 seconds (78–141 seconds), and the mean time in laughing seizures with crying was 96 seconds (69–132 seconds) in 4 patients. The seizures were reevaluated according to the rhythm of the day. Of the 128 seizures, 110 (86%) happened while the patient was awake and 18 (14%) while they were asleep. In the interictal EEG, 21 patients had epileptiform activities, 8 had non-epileptiform activities and 2 had both. Considering the anatomical distribution of interictal EEG findings, focal activity (class 1) was observed in 17 patients, regional activity (class 2) in 2 patients, generalized activity (class 3) in 5 patients, and multifocal activity (class 4) in 7 patients ( Table 1 ). Focal findings in 12 patients suggested temporal lobe localization.

**Table 1:** General features of patients with gelastic seizures and dacrystic attacks

	Gender/Age/Hand Dominance	Age of Seizure Onset (Years)	Semiology	Interictal EEG (EEG class)	Ictal EEG (EEG class)	MRI	PET-CT/MR	Surgery-pathology
**1**	**M/69/Right**	**16**	Laughter with mirth preserved awareness	F3-T5 slowing(1)	Left frontal spike (1)	Normal	Not performed	Not performed
**2**	**F/23/ Right**	**9 months**	Laughter without mirth, nystagmus	T4-C4-P4 and T3-C3-P3 sharp waves (2)	Artifacts (5)	Right precentral cortical dysplasia	Right parietal hypometabolism	cortical dysplasia
**3**	**M/42/ Right**	**1**	Smile, preserved awareness	F8, Fp2 sharp waves (1)	Fp1-F7, P3-O1 rhythmic theta (3)	Normal	Right frontotemporal hypometabolism	Not performed
**4**	**M/29/ Right**	**6**	Smile, fear	T3-F3-P3 sharp wave (2)	Left hemisphere spike waves (2)	Left Temporal Atrophy	Left temporal hypometabolism	Hippocampal sclerosis
**5**	**F/51/ Right**	**28**	Smile, Automatisms	F8 sharp wave, bilateral FIRDA (3)	F8-T4 rhythmic theta activity (1)	Right MTS	Right temporal hypometabolism	Not performed
**6**	**F/51/ Right**	**1 month**	Smile, bilateral tonic-clonic seizure	Bilateral FT focus (3)	P4-02-F8/F7T3 spikes (3)	Bilateral MTS	Bilateral temporal hypometabolism	Not performed
**7**	**M/48/ Right**	**2,5**	Smile, lack of awareness, automatisms	F7-T3/Fp2– F8 Bilateral focus (3)	F7-T3-T5-O1 rhythmic theta (3)	Left MTS	Left temporal hypometabolism	HS type I
**8**	**M/24/ Right**	**16**	Smile, automatisms	Fp1-F7 Sharp wave (1)	Bifocal FT sharp wave (3)	Right temporal tumor	Right temporal hypoperfusion	DNET
**9**	**M/43/ Right**	**23**	Laughter without mirth, lack of awareness	F8 spike (1)	F8 spike (1)	Right MTS	Not performed	Not performed
**10**	**M/22/ Right**	**21**	Laughter without mirth, lack of awareness	Left FT slow wave activity (1)	Artifacts (5)	Normal	Not performed	Not performed
**11**	**M/10/Left**	**14 months**	Laughter with mirth, bilateral tonic-clonic seizure	Generalized spike wave (4)	Bifrontal fast rhythm (3)	Normal	Not performed	Not performed
**12**	**M/17/Left**	**6**	Laughter with mirth, bilateral tonic-clonic seizure	F3-P3-F4 multifocal spikes (4)	F8 sharp-wave activity (1)	Diffuse hemispheric encephalomalaciain the left hemisphere	Not performed	Functional hemispherectomy
**13**	**M/30/Left**	**18**	Laughter with mirth	F8 sharp wave, F3-T5 spike (4)	F4 fast rhythm (1)	Atrophy at the left hemisphere	Left hemisphere hypometabolism	Not performed
**14**	**F/25/ Right**	**2**	Laughter with mirth	Generalized slow wave activity (4)	artifacts (5)	Hypothalamic Hamartoma	Bifronto, bitemporal hypometabolism	hamartoma
**15**	**M/45/ Right**	**13**	Smile, preserved awareness	F8 sharp waves-FIRDA (1)	F8-F4 rhythmic theta (1)	Right MTS	Right mesial temporal hypometabolism	FCD
**16**	**M/17/ Right**	**14**	Smile, preserved awareness	F7-T3 spike wave (1)	F7-C3, T3 semirhythmic delta waves (2)	Left MTS	Left insular hypometabolism	HS Type 1
**17**	**M/34/Left**	**5**	Laughter with mirth	Bi-frontal theta waves (3)	F4-F3 rhythmic activity (3)	Hypothalamic Hamartoma Right temporal cortical dysplasia	Right temporal hypometabolism	Hamartoma
**18**	**F/17/ Right**	**14**	Laughter with mirth	Bi-frontal spikes(3)	Bi-frontal spikes (3)	Normal	Not performed	Not performed
**19**	**M/36/ Right**	**21**	Laughter without mirth	Left FT theta waves (1)	F7-T3 sharpwave activity (1)	Diffuse cerebral atrophy	Left temporal hypometabolism	Not performed
**20**	**M/38/ Right**	**30**	Smile, lack of awareness Automatisms	F8-T4 spike wave activity (1)	F8-T4 spikewave activity (1)	Right temporal dysplasia	Left temporal hypometabolism	Cortical Dysplasia
**21**	**M/42/ Right**	**13**	Laughter with mirth, Automatisms	F7-T3 spike wave activity(1)	F7-T3 spikewave activity (1)	Normal	Bilateral temporal hypometabolism	HS Type-1
**22**	**F/37/ Right**	**8**	Laughter without mirth, lack of awareness	F7-T3 spike wave activity (1)	F7-T3 spikewave activity (1)	Normal	Normal	Not performed
**23**	**F/37/ Right**	**15**	Laughter without mirth	F7-T3 theta waves (1)	F7-T3 spike wave activity (1)	Normal	Normal	Not performed
**24**	**F/52/ Right**	**27**	Smile	F8-T4 spike wave activity (1)	F8 rhythmic theta waves (1)	Right temporal DNET	Right temporal hypoperfusion	DNET
**25**	**F/47/ Right**	**30**	Laughter without mirth	Diffuse slowing(4)	F8-T4 sharp wave activity (1)	Hypothalamic Hamartoma	Bifronto, bitemporo hypometabolism	Ganglion cell hamartoma
**26**	**F/34/ Right**	**20**	Smile, preservedawareness	F7-T3 spikewave activity (1)	F7-T3-T5 rhythmic theta waves (2)	Left MTS	Left mesial temporal hypometabolism	HS type I
**27**	**M/20/ Right**	**6 months**	Smile, preserved awareness	Multifocal multiple spike wave activity (4) (4)	Tonic pattern, F4-CZ semirhythmic delta waves (4)	Normal	Not performed	Not performed
**28**	**M/68/Right**	**12**	weeping loudly and Laughter without mirth	F8-T4 sharp wave activity (1)	F8 sharp wave (1)	Right MTS	Right temporal hypometabolism	Not performed
**29**	**F/23/ Right**	**16**	Crying- Laughter without mirth, bilateral tonic-clonic seizure	Diffuse theta (4)	Artifacts (5)	Cortical atrophy	Normal	Not performed
**30**	**F/18/ Right**	**9**	weeping loudly and Laughter without mirth, preserved awareness	Left frontal spikes (1)	Left frontal spikes (1)	Normal	Normal	Not performed
**31**	**F/28/ Right**	**20**	Crying- Laughter without mirth	F8-T4 sharp waves (1)	F8-T4 sharp waves (1)	Right temporal residual tumor	Not performed	Oligodendroglioma

Abbreviations: DNET, dysembryoplastic neuroepithelial tumors; FCD, focal cortical dysplasia; FIRDA, frontal intermittent rhythmic delta activity; HS, hippocampal sclerosis; MTS, mesial temporal sclerosis.

The ictal EEGs of four patients were inconclusive due to artefacts (class 5). Focal activity (class 1) was observed in 16 patients, regional activity (class 2) in 5 patients, and generalized activity (class 3) in 6 patients ( Table 1 ). The onset suggested the temporal lobe in 15 patients and the frontal lobe in 1 patient. In four patients with laughing/crying attacks, the ictal onset suggested the temporal lobe in two patients and the frontal lobe in one patient. A hypothesis concerning the ictal onset zone was not possible in 12 patients.

Cranial MRI findings were found to be normal in 10 patients, whereas 3 patients had findings consistent with cerebral atrophy, including 1 patient with unilateral hemispheric localization. Hypothalamic hamartoma was observed in two patients and one patient had findings consistent with unilateral dysplasia in the temporal cortex concomitant with hypothalamic hamartoma. In 13 patients, the MRI showed temporal localization. Cranial MRI revealed mesial temporal sclerosis in eight patients, including left mesial temporal sclerosis in three patients, right mesial temporal sclerosis in four patients, and bilateral temporal sclerosis in one patient. Considering extratemporal localization, hemispheric encephalomalacia was observed in one patient and frontal lesions were observed in another patient ( Table 1 ). Positron emission tomography-computed tomography/magnetic resonance imaging was performed on 24 patients. While 4 of these patients were found to be normal, 16 patients had focal hypometabolism, 4 patients had bilateral or multifocal hypometabolism, and PET could not be performed in 7 patients. ( Table 1 ). Fifteen patients underwent surgery for epilepsy. Six patients had findings consistent with hippocampal sclerosis. Hypothalamic hamartomas were classified as class 2 according to classification. 7 A stereoelectroencephalography (SEEG) exploration was conducted on the patient revealing hypothalamic hamartoma and focal cortical dysplasia, and all three patients with hypothalamic hamartomas were resected using a trans-Sylvian approach (patients 14, 17, and 25, Table 1 ). A patient underwent functional hemispherectomy (patient 12). In the postoperative period, 11 patients experienced follow-up as Engel class I and 4 patients as Engel class II ( Table 2 ).

**Table 2:** Features of operated patients

Patient numbers	Gender/age/hand dominance	Semiology	Engel classification	MRI	Pathology report	Operated area
2	F/23/Right	Laughter with mirth, awareness	Class-1	Right precentral cortical dysplasia	cortical dysplasia	Frontal lobe
4	M/29/Right	Smile, fear	Class-1	Left temporal atrophy	HS	Temporal lobe
7	M/48/right	Smile awareness, automatisms	Class-1	Left MTS	HS	Temporal lobe
8	M/24/right	Smile, automatisms	Class-2	Left temporal lesion	DNET	Temporal lobe
12	M/17/left	Laughter with mirth, bilateral tonic-clonic seizure	Class-2	Encephalomalacia in the left hemisphere		Frontal lobe-temporal lobe
14	F/25/right	Laughter with mirth	Class-1	Hypothalamic hamartoma	Hamartoma	Hypothalamus
15	M/45/right	Smile, awareness	Class-1	Right MTS	FCD	Temporal lobe
16	M/17/right	Smile, awareness	Class-1	Left MTS	HS	Temporal lobe
17	M/34/left	Laughter with mirth	Class-1	Hypothalamic hamartoma	Hamartoma	Hypothalamus
20	M/38/right	Smile, awareness, automatisms	Class-2	Right temporal dysplasia	FCD	Temporal lobe
21	M/42/right	Laughter with mirth, automatisms	Class-1	Normal	HS	Temporal lobe
24	F/52/Left	Smile	Class-1	Right temporal DNET	DNET	Temporal lobe
25	F/47/Right	Laughter without mirth	Class-1	Hypothalamic hamartoma	Hamartoma	Hypothalamus
26	F/34/Right	Smile, awareness	Class-1	Left MTS	HS	Temporal lobe
31	F/28/right	Crying- laughter without mirth	Class-2	Right temporal tumor	Oligodendroglioma	Temporal lobe

Abbreviations: DNET, dysembryoplastic neuroepithelial tumors; FCD, focal cortical dysplasia; HS, hippocampal sclerosis; MRI, magnetic resonance imaging; MTS, mesial temporal sclerosis.

### Features according to subgroups of gelastic seizures

Overall, 128 seizures were evaluated. Of the 27 patients, 12 had seizures that manifested with smiling, 7 had seizures that manifested with laughing with mirth and 8 had seizures that manifested with laughter without mirth. Both laughing and crying episodes were observed in four patients, while two patients had crying and laughter without mirth and two patients had weeping loudly and laughter without mirth episodes. Ten patients presented 38 seizures without additional symptoms (4 patients with laughter with mirth, 3 with laughter without mirth, 1 smile, 1 weeping loudly/laughter without mirth, and 1 crying/laughter without mirth). Additional ictal signs were observed in 14 seizures of 10 patients, which evolved to bilateral tonic-clonic seizures following 1 laughter with mirth, 3 laughter without mirth and 5 smiles ( Table 1 ). In four of the eight patients who exhibited laughter with mirth, the MRI examination was normal, and one had unilateral cerebral atrophy. Hypothalamic hamartoma was observed in two patients. One patient had diffuse encephalomalacia in the left hemisphere, and the ictal EEG findings were limited to the frontal electrodes (Patient 12). Magnetic resonance imaging examination was normal in four patients who exhibited laughing without mirth. One patient had a hypothalamic hamartoma. Frontal cortical dysplasia (FCD) with frontal localization was observed in one patient, and a temporal lobe lesion was present in one. Twelve patients had seizures that manifested with smiling. MRI examinations were normal in two of these patients, and the other patients had epileptogenic lesions within the temporal lobe. Nine of the patients underwent temporal lobectomy for epilepsy ( Table 2 ). Milder seizures persisted in three of these patients without any emotional components (Patients 8, 20, and 31). Laughing was observed in all patients who had seizures accompanied by crying. While the collected data suggested the temporal lobe in two of these patients and the ictal data suggested the temporal lobe in one patient, it was not possible to predict the localization with available data in the other patient ( Table 1 ).

## DISCUSSION

In 1971, Gascon and Lombroso stated that recurrent laughing attacks could accompany an epileptic event, and they emphasized that gelastic epilepsy should be considered in patients who have abnormal ictal or interictal EEG findings, as well as stereotypic and repeating laughing attacks. 7 Laughing attacks are quite rare, short-lasting, unprovoked, and uncontrollable bouts of laughing. 1

The neural correlates of physiological laughing involve the amygdala, thalamic/hypo and subthalamic areas, and the dorsal/tegmental brainstem. A second voluntary system originates in the premotor/frontal opercular areas and leads through the motor cortex and pyramidal tract to the ventral brainstem. These systems and the laughter response appear to be coordinated by a laughter-coordinating center in the dorsal upper pons. For the perception of humor, the right frontal cortex, the medial ventral prefrontal cortex, the right and left posterior (middle and inferior) temporal regions and possibly the cerebellum seem to be involved to varying degrees. 8 9 Gelastic seizures can be observed as a result of frontal, temporal, limbic, insular, and hypothalamic lesions, in addition to the spread of ictal discharges that stem from the primary lesion to other cortical areas that control laughing, regardless of the cause. 10 Although laughing seizures do not originate from a certain anatomical region according to recent studies, they can start from the temporal, parietal or frontal lobe, as well as from hypothalamic hamartomas originating primarily from the hypothalamus in children; even non-lesional cases have been reported. 3 4 Iapadre et al. shared data from 30 pediatric patients who had gelastic seizures without hypothalamic hamartoma. Nineteen of these children did not have any lesions, whereas 11 children had lesions. It was asserted that the group without lesions responded better to drug treatment. In the ictal EEG examination of these patients, frontal or temporal localization was noted in both groups. 4 However, cases with parietal focus have also been reported in the literature. 11

**Figure 1:**
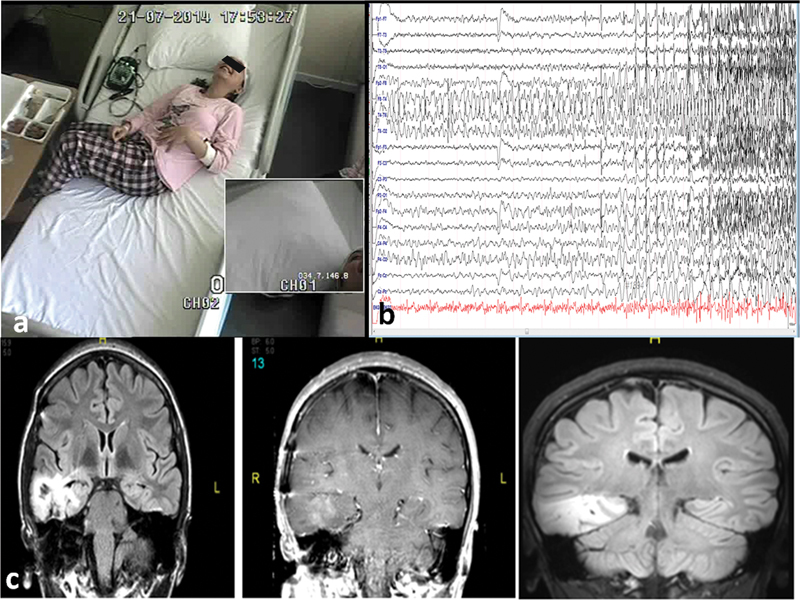
Patient 31: a) Crying-laughter without mirth, b) ictal EEG: F8-T4 sharp waves, c) cranial MRI, right temporal residual tumour.

**Figure 2:**
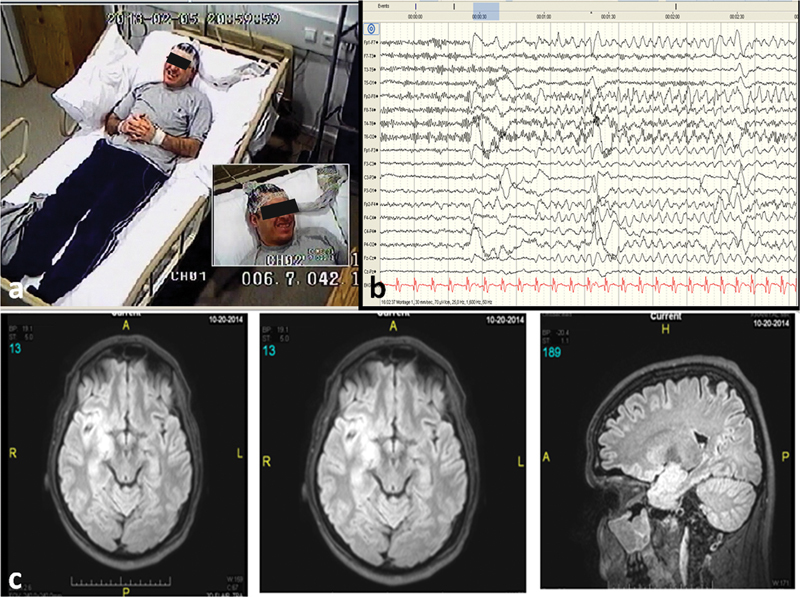
Patient 20: a) Smile, awareness and automatisms, b) ictal EEG: F8-T4 spike wave activity, c) cranial MRI, right temporal dysplasia.

It seems that there is no single zone responsible for the perception of humor and laughter, and, therefore, gelastic seizures also seem to originate from widespread cortical areas, probably a neural network involving the temporal and frontal lobes. It was also claimed that the symptomatogenic zone was not the hamartoma in a case of laughter observed with hypothalamic hamartomas. 3 Here, we question which neural circuits cause gelastic seizures and laughing during seizures. According to the records of a group of patients with hypothalamic hamartoma obtained from electrodes located on both the hamartoma and cortical area, it was underlined that from a topographic aspect, gelastic seizures were associated with spread to the orbitofrontal areas, the cingulate cortex and neighboring limbic structures. In seizures with automatisms in which awareness is affected, the spread was more towards the mesial temporal structures. 12 From a semiological point of view, gelastic seizures are classified as emotional seizures. A case report of a patient with frontoopercular dysplasia extending to the posterior insula was evaluated by scalp and intracranial electrodes and revealed no symptomatogenic zone for gelastic and dacrystic behaviors. While the patient was rendered seizure-free by resection and laser ablation of the dysplastic cortices, the authors discussed that gelastic and dacrystic semiologies represent ictal automatisms, and their expression results from the interaction of cortical and subcortical networks. They added that automatisms may be provoked by cortical-subcortical loops involving the limbic pathways. However, the International League Against Epilepsy (ILAE) proposes classifying these seizures as focal nonmotor-onset emotional seizures. 2 However, as in the literature and this case study, at least in a group of these seizures, there are no emotions, rather only motor behavior of laughing or crying. Therefore, it is debatable from the nosological perspective whether these phenomena represent emotions or automatisms. Mirth is an important element of gelastic seizures. Classically, seizures are classified according to the presence or absence of accompanying mirth. It is questionable whether the existence of mirth indicates localization. Based on the surgically treated cases, the neural correlates of laughter without mirth or, in other words, the motor act of laughter was supposed to be the anterior cingulate gyrus; on the other hand, the basal temporal cortex may contribute to emotional content. 9 13 Electrical stimulation of the right anterior cingulate gyrus elicited smiles and laughter but not mirth, and, at low voltages, smiling was contralateral, indicating the role of the anterior cingulate gyrus in the motor function of laughter. 14 Patients who are conscious during laughing attacks report no emotion or motivation for laughing. 10 15 16 17 18 In our case series, it was observed that the patients in whom ictal activity spread to the frontal region (those with temporal lobe lesions) showed an expression of laughing. Only the right-handed-dominant patients had mesial temporal sclerosis, one of which had the bilateral localization component but not the emotional component ( Table 1 ; Figure 1 a, b , and c [patient 31]; Figure 2 a, b , and c [patient 20]), which contributes to the above argument. However, in our study, the origin of laughter with and without mirth showed diverse localizations, but not all patients in our study were evaluated and resected in terms of epilepsy surgery, making a definite conclusion possible.

Molinuevo and Arroyo saw a total of 39 seizures in 5 patients. Twenty-nine of these had seizures accompanied by smiling (74%), and other seizures were complex partial or simple partial seizures without smiling. 19 In our sample, we found 60% (128/212) and this group is more homogenous concerning localization. Cranial MRIs revealed lesions in the temporal lobe in 10 of 12 patients, 9 of whom underwent temporal lobectomy with a favorable outcome ( Table 2 ). Although it is hard to propose a single symptomatogenic zone for gelastic seizures, generally, we can speculate that smiling seizures or their emotional contribution come from the temporal lobes. The patients who exhibited laughing accompanied by crying, which was less frequent than the former groups, were included as the fourth group. Patients with gelastic and coexisting dacrystic seizures had lesions in different anatomical locations and of different etiologies.

According to an extensive study, the prevalence of dacrystic seizures was 0.1% among patients who underwent video-EEG monitoring. 20 It was noted that the seizures originated from the frontal lobe in two of these five patients, and the frontal lobe was suspected in the other two patients. In another study of 11 patients with dacrystic seizures, there was no conclusive data for lateralization Dacrystic seizures have not been investigated in a high number of studies; therefore, lateralization and localization values are unknown.

In light of these data, it can be argued that in this series of patients, the symptom that manifested as a smile mainly originated from the temporal lobe structures. We may speculate that temporal lobes contribute to the emotional part of a laugh and that no single zone can be defined as the major symptomatogenic zone for this complex, high-order behavior.
